# Handheld imageless robotic total knee arthroplasty improves accuracy and early clinical outcomes when compared with navigation

**DOI:** 10.1186/s42836-025-00303-4

**Published:** 2025-04-04

**Authors:** Joshua Yeuk-Shun Tran, Abbie Yan-Tung Tang, Cham-Kit Wong, Gloria Yan-Ting Lam, Tsz-Lung Choi, Rex Wang-Fung Mak, Jonathan Patrick Ng, Kevin Ki-Wai Ho, Michael Tim-Yun Ong, Patrick Shu-Hang Yung

**Affiliations:** 1 Department of Orthopaedics and Traumatology, Chinese University of Hong Kong, Hong Kong SAR, China; 2 Department of Orthopaedics & Traumatology, Alice Ho Miu Ling Nethersole Hospital, Hong Kong SAR, China; 3 Department of Orthopaedics and Traumatology, Prince of Wales Hospital, Hong Kong SAR, China; 4 Department of Orthopaedics and Traumatology, CUHK Medical Centre, Hong Kong SAR, China

**Keywords:** Handheld robotic surgery, Total knee arthroplasty, Imageless navigation, Surgical accuracy, Early clinical outcomes, Robotic-assisted surgery, Accelerometer-based navigation

## Abstract

**Background:**

This study compared imageless robotic-assisted total knee arthroplasty (RATKA) with accelerometer-based navigation (ABN) systems in terms of surgical accuracy and early clinical outcomes.

**Methods:**

A retrospective analysis was conducted on 153 patients (178 knees) who had undergone primary TKA from 2017 to 2023. Surgical accuracy and functional outcomes were assessed up to 12 months post-operation using the Chi-square test, Student’s *t*-test, and ANCOVA. Subgroup analyses based on patient demographics were also conducted.

**Results:**

Among 153 patients, 101 underwent RATKA, and 52 received ABN. RATKA demonstrated superior alignment accuracy with a significantly lower deviation from the planned alignment (*P* < 0.05). Additionally, RATKA led to significantly better postoperative functional scores at 6 weeks (*P* = 0.001) and 3 months (*P* = 0.001), even after adjusting for preoperative functional differences.

**Conclusions:**

RATKA offers enhanced precision and improves early recovery compared to ABN, supporting its potential as a preferred technology for TKA. Its ability to optimize kinematic alignment may contribute to superior patient outcomes. Compared to ABN, RATKA provides a unique advantage by achieving greater accuracy in planned alignment, which may translate into improved functional recovery. Further research with larger cohorts is recommended to confirm these findings.

**Supplementary Information:**

The online version contains supplementary material available at 10.1186/s42836-025-00303-4.

## Introduction

Total knee arthroplasty (TKA) is a widely performed procedure for pain relief and functional restoration in degenerative joint disease, with the long-term survival being longer and global demand on the rise [1, 2]. By 2030, TKA procedures are projected to grow by 85% (1.26 million procedures) globally, with U.S. demand expected to rise by 139% by 2040 and 469% by 2060, while the U.K. demand is estimated to increase by 117% by 2030 [3, 4]. Despite these advancements, achieving optimal alignment remains a significant challenge, as malalignment is associated with polyethylene wear, prosthetic loosening, and increased failure rates [5]. Furthermore, patients with alignment outliers—such as distal femoral angle (DFA), proximal tibial angle (PTA), or posterior slope angle (PSA)—report worse functional outcomes and greater dissatisfaction compared to non-outliers [6]. Despite improvements in surgical techniques and surgeon expertise, alignment outliers persist at high rates, which continue to impact patient satisfaction and long-term implant survival [7].

To enhance alignment accuracy, computer-assisted TKA technologies have been developed to improve the accuracy of the implant position and alignment, categorized into passive, semi-active, and active systems [8]. Passive systems provide intraoperative guidance requiring full surgeon control, while semi-active systems offer real-time feedback and robotic assistance to enhance precision with surgeon control, and active systems autonomously perform bone resections without direct surgeon involvement [9]. Robot-assisted TKA (RATKA) systems, such as NAVIO and CORI, fall under the semi-active category and employ intraoperative registration and robotic guidance to refine bone resections and optimize soft tissue balancing [10]. Accelerometer-based navigation (ABN) systems, such as KneeAlign 2, are passive systems that rely on position sensors to guide bone resections and assess alignment without preoperative imaging [11].

Recent studies have compared RATKA and ABN with conventional techniques for TKA. RATKA has shown improved postoperative alignment and clinical outcomes compared to conventional methods [12]. Similarly, ABN demonstrated higher rates of neutral alignment postoperatively [13, 14]. However, limited research has directly compared their impact on early functional outcomes and surgical accuracy.

This study aimed to address this gap by evaluating and comparing the early functional outcomes and surgical accuracy of semi-active imageless RATKA and passive imageless ABN in primary TKA, seeking to provide insights into their relative effectiveness to optimize TKA techniques and improve patient outcomes.

## Materials and methods

### Study design

This was a retrospective analysis of prospectively collected data in the authors’ institutional joint registry. Ethical approval was obtained from the Institutional Ethics Review Committee of the Joint CUHK-NTEC Clinical Research Ethics Committee.

### Study population

This study included female and male patients over 40 years old who suffered from end-stage osteoarthritis of the knee (Kellgren and Lawrence grade 3–4) and underwent navigation or robotic-assisted primary total knee replacement from 2017 to 2023. Patients were excluded if they had undergone previous surgery on the same knee, including previous knee arthroplasty or osteotomy or had underlying diseases or abnormal anatomy complicating the surgery, including previous periarticular fracture, severe fixed flexion contracture > 20°, multi-ligament instability, bone stock deficiency requiring augmentation and stems, neuromuscular disorder, acute and chronic infections.

### Intervention

All surgeries were performed in a tertiary referral hospital and its affiliated joint replacement center by the same team of experienced specialist orthopedic surgeons from the arthroplasty division. The experienced arthroplasty surgeons performed at least 20 TKA with navigation or robotic system before the start of the recruitment. Comparable experience with the use of the two systems was ensured.

Two navigation systems were utilized in the study: Total knee replacement with a Hand-held accelerometer-based navigation (ABN) system KneeAlign 2 (KA2) (OrthAlign, Inc.; Aliso Viejo, CA, USA) and total knee replacement with a NAVIO or CORI robot (Smith and Nephew, USA). The choice of systems was based on the availability of the systems in the hospital at the time of surgery. All TKAs were performed using either kinematic alignment (KA) or mechanical alignment (MA) according to the surgeons’ preference. Surgical techniques were standardized. All TKAs were performed via a standard medial para-patellar approach with measured resection technique, soft tissue balancing, and then implantation of components with antibiotics-loaded cement. Implants used were either Legion or Journey II BCS total knee system (Smith & Nephew, plc.; Watford, UK). In the KA2 group, the system was employed for proximal tibial and distal femoral osteotomy. In the robotic group, mapping was done and the bone cutting was completed using a combination of burrs and saws.

All patients received identical wound closure techniques and postoperative recovery protocol (adult joint reconstruction enhanced recovery after surgery perioperative analgesic & antiemesis protocol). All patients followed the standard physiotherapy adult joint reconstruction rehabilitation protocol. Patients were discharged from the hospital once their mobility allowed for outpatient care.

### Radiographic analysis

**Fig. 1:**
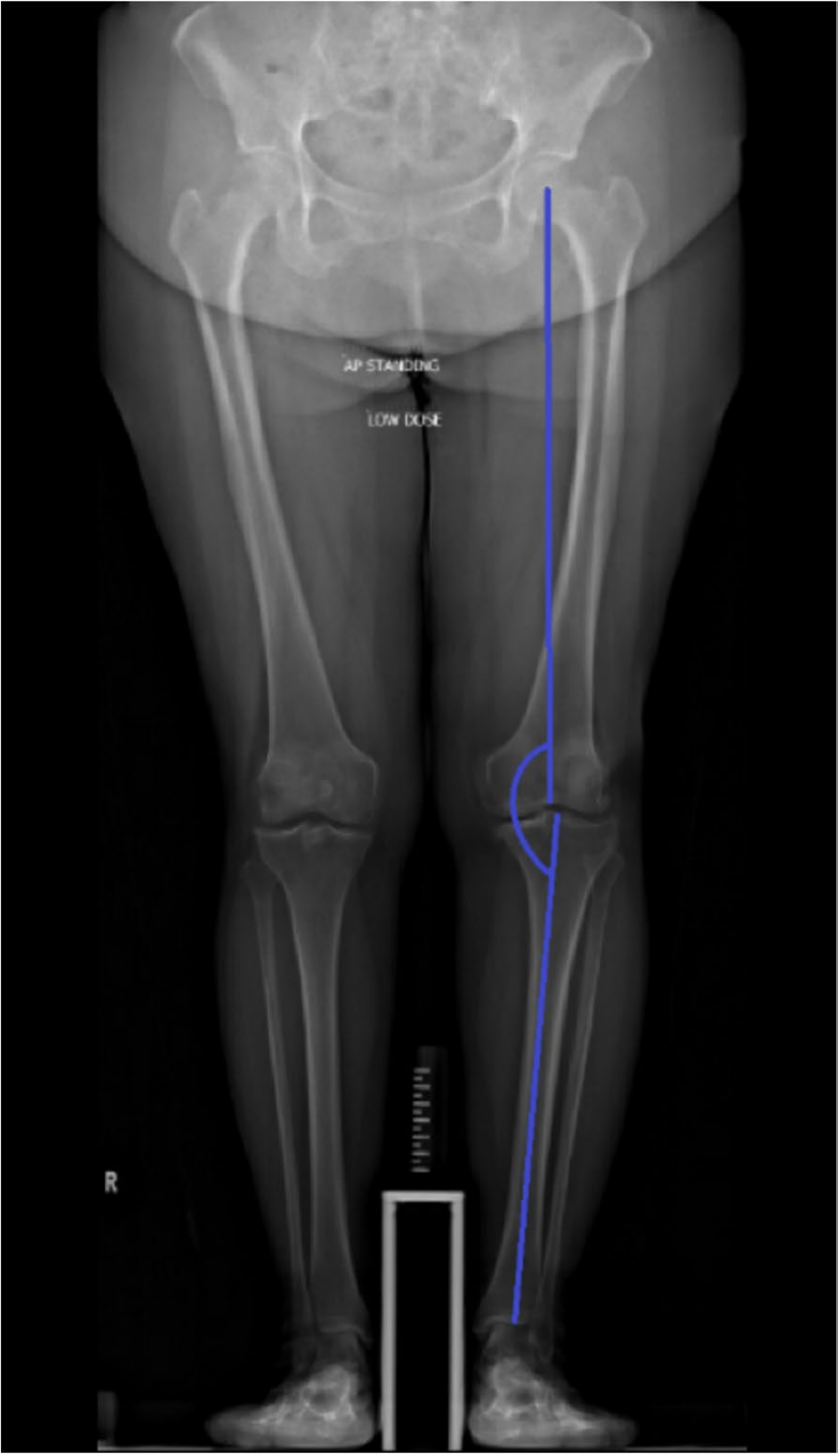
Measurement of hip knee ankle (HKA) angle

Preoperative and postoperative anterior–posterior (AP) standing long-leg radiographs were reviewed by two independent reviewers who were blinded to the intervention grouping. Measurements of hip knee ankle angle (HKA) were performed manually using digital measurement tools. HKA measures the angle between the mechanical axis lines of the femur (from the femoral head center to the intercondylar fossa center) and of the tibia (from the tibial interspinous groove to the tibial mid-plafond). The measurements are further detailed in Fig. 1.

The difference between planned alignment and measured alignment (postoperative HKA) is calculated by subtracting the planned angle from the postoperative HKA. A positive difference indicates a valgus angulation of the knee, while a negative difference is indicative of varus angulation. The absolute difference is then obtained by converting the negative values into positive values to obtain the accuracy of alignment.

### Power analysis

A G*Power analysis was conducted based on the sample size. An independent *t*-test was selected with a post hoc power analysis to compute achieved power. Given a two-tailed input, an effect size of 0.5, and an α of 0.05, the calculated power was 0.8933.

### Statistical analysis

Demographic data (age, sex, BMI, degree of varus deformity, range of motion, and the side of operation) were compared between the ABN group and the RATKA group using Student’s *t*-test or Chi-square test. Operative parameters, including operating time, tourniquet time, pressure, and blood loss, were extracted. Knee Society Knee Score (KSS) and Knee Society Function Score (KSFS) at preoperation, 6 weeks, 3 months, 6 months, and 12 months post-operation were compared between the two groups using Student’s *t*-test. Pre- and postoperative HKA were compared between the two groups using Student’s *t*-test. ANCOVA was then used to remove the effects of covariates, including preoperative functional scores between patients when comparing postoperative data between the two groups. Further subgroup analysis based on age, BMI, and sex was done to stratify the results. Inter-rater reliability was assessed to compare radiological measurements in terms of two-way random effects model Cronbach α values (Supplementary Information: Table S1). All statistical analyses were carried out using IBM SPSS version 28 (Armonk, NY: IBM Corp). Statistical significance was set at *P* < 0.05.

## Results

A total of 153 patients (178 knees) were recruited into the study. 101 patients (112 knees) were in the robotic group, while 52 patients (66 knees) were in the navigation group. The preoperative patient demographics between the robotic and navigation groups were generally comparable (Table 1). Significant differences between the preoperative functional scores were observed for both the KSFS (*P* < 0.001) and KSS (*P* < 0.001). Other operative parameters are also shown (Supplementary Information: Table S2).

**Table 1:** Patient demographics

	Robotic-assisted (*n* = 112)	ABN (*n* = 66)	*P*-value (95% CI)
Mean ± SD			
Age	70.8 ± 7.1	69.1 ± 7.6	0.121 (−4.0, 0.5)
BMI	27.2 ± 3.8	26.7 ± 3.0	0.356 (−1.6, 0.6)
ROM	99.5 ± 19.4	94.2 ± 18.3	0.071 (−11.2, 0.5)
KSFS Preop	58.7 ± 18.6	38.8 ± 17.6	0.001 (−15.8, −4.0)*
KSS Preop	58.2 ± 20.3	37.4 ± 14.0	0.001 (−26.7, − 15.0)*
*n* (%)			
Sex (female)	84 (75.0%)	39 (45.3%)	0.027*
Side (left)	53 (47.3%)	33 (38.4%)	0.730

^*^P < 0.05, statistically significant

The alignment was significantly more accurate in the robotic group than in the navigation group (*P* < 0.05) when comparing the planned alignment with the postoperative HKA in terms of absolute difference (Table 2). An absolute difference approach was used to evaluate the degree of variance between the planned alignment and postoperative HKA, eliminating the influence of varus or valgus variance. Moreover, the robotic group showed significantly better patient functional outcomes relative to the navigation group in terms of KSFS at 6 weeks (*P* = 0.001) and 3 months (*P* < 0.01).

**Table 2:** Postoperative functional scores—Student’s *t*-Test

	Robotic-assisted (*n* = 112)	ABN (*n* = 66)	*P*-value (95% CI)
Mean ± SD			
KSFS 6 Weeks	60.7 ± 18.6	46.9 ± 26.9	0.001 (−22.0, −5.7)*
KSS 6 Weeks	88.3 ± 9.8	59.5 ± 7.3	0.457 (−1.8, 4.1)
KSFS 3 Months	75.3 ± 14.4	65.3 ± 21.7	0.004 (−16.8, −3.3)*
KSS 3 Months	91.5 ± 9.7	92.1 ± 6.1	0.326 (− 2.2, 3.6)
KSFS 6 Months	78.4 ± 16.3	78.6 ± 17.3	0.845 (−5.7, 6.1)
KSS 6 Months	93.7 ± 8.3	94.7 ± 6.0	0.474 (−1.7, 3.6)
KSFS 12 Months	82.5 ± 14.7	77.2 ± 22.9	0.102 (−11.8, 1.1)
KSS 12 Months	95.4 ± 5.2	96.5 ± 4.5	0.186 (−0.5, 2.7)
Difference	−0.74 ± 2.02	−0.39 ± 3.31	0.193 (−0.4, 1.1)
Absolute Difference	1.69 ± 1.32	2.30 ± 2.39	0.029 (0.1, 1.2)*

^*^P < 0.05, statistically significant

Further analysis was done to account for the preoperative functional differences between the robotic and navigation groups (Table 3). After controlling for the preoperative functional scores, the robotic group still showed significantly better early postoperative functional outcomes in terms of KSFS at 6 weeks (*P* < 0.001) and 3 months (*P* = 0.025). Although ANCOVA was used, the significant differences in the preoperative functional scores introduced confounders to the data.

**Table 3:** Preoperative and postoperative functional scores—ANCOVA

	Robotic-assisted (*n* = 112)	ABN (*n* = 66)	*P*-value
Mean ± SD			
KSFS Pre-Op	58.7 ± 18.6	38.8 ± 17.6	
KSFS 6 Weeks	60.7 ± 18.6	46.9 ± 26.9	< 0.001*
KSFS 3 Months	75.3 ± 14.4	65.3 ± 21.7	0.025*

^*^P < 0.05, statistically significant

Further subgroup analysis was performed to stratify the patient groups based on age, BMI, and sex (Supplementary Information: Tables S3–S5). 65 or above was selected as the cut-off for the elderly population as widely defined in the locality. 25 or above was taken as the cut-off for obesity as widely defined in Asian populations.

## Discussion

The current study demonstrated that RATKA systems attained significantly better alignment accuracy and early postoperative functional outcomes when compared to ABN systems. Although studies have shown that RATKA or ABN systems significantly improved alignment accuracy and functional outcomes when compared to conventional TKA, there is limited evidence based on direct comparisons between RATKA and ABN systems.

Handheld imageless systems offer several advantages over their image-based counterparts. The reduced need for preoperative imaging lowers the utilization rate of hospital computed tomography (CT) scan [17]. This increases efficiency and allows for better patient selection for CT scans, improving patient flow. Furthermore, the elimination of the preoperative CT scan reduces radiation exposure of patients [15]. Importantly, the learning curve of handheld imageless systems has shown to be comparable to that of existing TKA methods [16]. Consequently, it is important to compare different imageless systems to improve patient outcomes.

RATKA systems are likely to have greater accuracy than navigation systems in bone resection, prosthesis positioning, and gap balancing due to their semi-active nature [17]. While the surgeon has overall control over osteotomy and prosthesis positioning, the system limits the range of movements of the surgical instruments according to the surgeon’s surgical plan [18]. Moreover, the robotic system can potentially augment the surgeon's skills by providing real-time visual, tactile, and auditory feedback for more precise execution of the planned cuts [19]. It was demonstrated that 91.6% of bone resections were within ≤ 1 mm of the preoperative plan in RATKA [17]. Another study also found the accuracy of RATKA in bone resection was high, with 99 of 105 bone resections being within 1 mm of the preoperative plan [20]. In contrast, ABN systems can only provide feedback and warning during the operation without restricting the surgeon [18].

Better accuracy in bone resection in RATKA, as shown in the study, allows kinematic alignment to be performed better. Proper KA could minimize the anatomical change of the bone and the impact on ligament balancefor better implant survival and functional outcomes [21, 22]. This highlights the possible importance of accurate bone resection following a preoperative surgical plan for TKAs.

TKA conducted with RATKA systems may also allow for earlier postoperative recovery. Studies have shown that RATKA results in shorter postoperative lengths of stay due to reduced blood loss [23, 24]. Accurate surgical planning with fewer bone cuts and less soft tissue management along with avoiding instrument insertion that reduces microembolus formation, could all contribute to the reduction in blood loss [24–26]. Reduced blood loss could thus reduce the need for transfusion and other associated complications, such as infection, venousthrombolism, and mortality [27]. RATKA is further associated with reduced postoperative pain, opiate analgesia requirements in the early postoperative period, and shorter time to discharge [23].

The benefits of RATKA for early functional outcomes were demonstrated in terms of KSFS at 6 weeks and 3 months. Previous studies found that RATKA sped up the early postoperative functional recovery time, allowing for early mobilization of patients [23, 28]. Early mobilization signifies earlier initiation of physical therapy for patients to train muscle strength and flexibility [29]. Starting rehabilitation within 24 h of TKA could result in greater joint range of motion, improved quadriceps and hamstring strength, and better scores for gait and balance [30]. A study showed that the early rehabilitation group had a better Western Ontario and McMaster Universities Osteoarthritis Index than the standard rehabilitation group at each subsequent visit [31].

Apart from the clinical benefits, the cost-effectiveness of RATKA and ABN is another consideration. A study showed that, compared to conventional standard, imageless handheld RATKA (NAVIO) required a 25-min more surgical time and resulted in an additional cost of USD 2600, while the cost was only USD 650 for imageless navigation [32]. While the surgical time is significantly longer for RATKA (117.44) than navigation (113.33 min), there were no differences in the length of stay, 90-day all-cause revisions, and 1-year PROM scores [33]. This suggests that the navigation system may be more cost-effective. Nevertheless, RATKA may be a cost-effective procedure at high-volume hospitals with a volume of more than 49 procedures per year [34]. This indicates that RATKA is potentially more cost-effective at high-volume hospitals while ABN is more beneficial for low-volume hospitals. Studies on direct comparisons of cost-effectiveness between RATKA and ABN are needed to further help identify suitable candidates for RATKA or ABN.

This study has several limitations. Firstly, the retrospective nature of this study limited the control over sampling and the potential effects of confounding factors. The differences in patient demographics introduced significant confounders to the study. Despite using ANCOVA, the power of the study may be impaired. Patellar resurfacing was not performed in all patients, while 16 studies of 1989 knees showed a significant difference in the functional score for ≥ 5 years between resurfacing and non-resurfacing in TKA [35]. The comparability of surgeons’ experience with RATKA and ABN systems should also be considered due to the learning curve. As a single-center study, the population was more homogenous, and the interventions were tested in relatively optimal conditions, while in the “real-world” setting, TKA is performed in patients with clinical and anatomical differences. Along with the small sample size of 178 patients, the external generalizability is therefore limited. Furthermore, limited surgeon experience may also reduce generalizability due to the learning curve of the systems. This study also has low comparability due to the lack of a conventional TKA group to provide a benchmark for evaluating if the differences in functional outcomes can be attributed to the interventions. As a result, the treatment outcomes may be over- or underestimated. Furthermore, this study showed no significant differences in functional outcomes after 3 months, which raises concern about the long-term benefits of RATKA. Therefore, prospective multi-center research comparing conventional TKA with RATKA and ABN with a larger sample size and longer follow-up periods should be conducted to validate the findings and guide clinical decision-making.

## Conclusion

In summary, the improved accuracy and early postoperative functional outcomes of RATKA may implicate it as the preferred technology for knee arthroplasties.

## Supplementary Information

Supplementary Material 1. Table S1. Cronbach α values, Table S2. Operative Parameters, Table S3. Subgroup Analysis Age – Student’s T-Test.

## Data Availability

Data are available on request due to privacy/ethical restrictions.
