# Supplementary material for: Handheld imageless robotic total knee arthroplasty improves accuracy and early clinical outcomes when compared with navigation
Source: Arthroplasty. 2025 Apr 4;7:18. doi: 10.1186/s42836-025-00303-4 (PMC11969756; doi:10.1186/s42836-025-00303-4)
Supplement: Supplementary file 1 — Supplementary Material 1. Table S1. Cronbach α values, Table S2. Operative Parameters, Table S3. Subgroup Analysis Age – Student’s T-Test. [file 42836_2025_303_MOESM1_ESM.docx]

**Supplementary Tables**

Supplementary Table 1. Cronbach α values

|  | Cronbach α (95%) |
| --- | --- |
| Pre-Operative HKA | 0.918 (0.856-0.953) |
| Post-Operative HKA | 0.846 (0.729-0.912) |

Supplementary Table 2. Operative Parameters

|  | Robotic-Assisted (n=112) | ABN (n=66) |
| --- | --- | --- |
| Mean ± SD |  |  |
| Operation Time | 125.9 ± 28.3 | 116.9 ± 22.8 |
| Tourniquet Time | 97.1 ± 19.0 | 86.0 ± 16.9 |
| Tourniquet Pressure | 280.3 ± 7.0 | 298.5 ± 5.3 |
| Blood Loss | 5.8 ± 15.5 | 5.2 ± 13.1 |

Supplementary Table 3. Subgroup Analysis Age – Student’s T-Test

|  | Robotic-Assisted | ABN | P-value (95% CI) |
| --- | --- | --- | --- |
| Adult - Mean ± SD | (n=21) | (n=17) |  |
| KSFS 6 Weeks | 62.4 ± 21.3 | 53.9 ± 26.0 | 0.329 (-25.8, 8.9) |
| KSS 6 Weeks | 88.2 ± 8.7 | 89.5 ± 5.2 | 0.660 (-4.6, 7.2) |
| KSFS 3 Months | 81.4 ± 11.1 | 73.0 ± 15.4 | 0.080 (-17.8, 1.0) |
| KSS 3 Months | 93.0 ± 6.8 | 92.1 ± 7.0 | 0.700 (-5.8, 4.0) |
| KSFS 6 Months | 84.7 ± 14.5 | 82.4 ± 17.5 | 0.672 (-13.5, 8.8) |
| KSS 6 Months | 90.6 ± 14.8 | 93.7 ± 7.7 | 0.447 (-5.2, 11.5) |
| KSFS 12 Months | 91.1 ± 12.3 | 84.4 ± 15.9 | 0.168 (-16.2, 2.9) |
| KSS 12 Months | 94.5 ± 8.0 | 96.4 ± 4.2 | 0.376 (-2.5, 6.3) |
| Difference | -1.10 ± 2.2 | 0.24 ± 3.2 | 0.133 (-0.4, 3.1) |
| Absolute Difference | 1.86 ± 1.5 | 2.24 ± 2.2 | 0.548 (-0.9, 1.7) |
| Elderly - Mean ± SD | (n=91) | (n=49) |  |
| KSFS 6 Weeks | 60.3 ± 18.0 | 44.6 ± 27.2 | 0.001 (-25.1, -6.3)* |
| KSS 6 Weeks | 88.4 ± 9.9 | 89.4 ± 7.9 | 0.543 (-2.4, 4.6) |
| KSFS 3 Months | 73.0 ± 15.0 | 62.2 ± 23.2 | 0.014 (-19.3. -2.2)* |
| KSS 3 Months | 90.9 ± 10.6 | 92.1 ± 5.8 | 0.485 (-2.3, 4.8) |
| KSFS 6 Months | 76.8 ± 16.7 | 76.7 ± 17.2 | 0.977 (-7.1, 6.9) |
| KSS 6 Months | 94.5 ± 5.6 | 95.1 ± 5.1 | 0.565 (-1.6, 2.9) |
| KSFS 12 Months | 80.1 ± 14.6 | 74.6 ± 24.6 | 0.165 (-13.5, 2.3) |
| KSS 12 Months | 95.7 ± 4.1 | 96.6 ± 4.7 | 0.311 (-0.8, 2.5) |
| Difference | -0.66 ± 1.98 | -0.61 ± 3.37 | 0.929 (-1.0, 1.1) |
| Absolute Difference | 1.65 ± 1.28 | 2.33 ± 2.49 | 0.079 (-0.1, 1.4) |

Supplementary Table 4. Subgroup Analysis BMI – Student’s T-Test

|  | Robotic-Assisted | ABN | P-value (95% CI) |
| --- | --- | --- | --- |
| Not Obese - Mean ± SD | (n=31) | (n=18) |  |
| KSFS 6 Weeks | 57.1 ± 19.7 | 62.7 ± 19.3 | 0.390 (-7.4, 18.6) |
| KSS 6 Weeks | 90.7 ± 6.1 | 89.5 ± 5.7 | 0.557 (-5.0, 2.7) |
| KSFS 3 Months | 77.1 ± 14.0 | 70.4 ± 22.9 | 0.325 (-20.4, 7.0) |
| KSS 3 Months | 92.6 ± 10.1 | 94.1 ± 3,6 | 0.588 (-4.2, 7.3) |
| KSFS 6 Months | 79.0 ± 20.3 | 80.0 ± 20.6 | 0.871 (-11.8, 14.0) |
| KSS 6 Months | 95.3 ± 5.7 | 95.0 ± 4.8 | 0.894 (-4.0, 3.5) |
| KSFS 12 Months | 83.5 ± 14.8 | 77.2 ± 28.8 | 0.410 (-21.7, 9.1) |
| KSS 12 Months | 97.4 ± 2.7 | 96.6 ± 4.1 | 0.441 (-3.0, 1.3) |
| Difference | -0.29 ± 1.68 | -0.28 ± 3.98 | 0.990 (-2.0, 2.1) |
| Absolute Difference | 1.26 ± 1.12 | 2.83 ± 2.73 | 0.030 (0.2, 3.0)* |
| Obese - Mean ± SD | (n=81) | (n=47) |  |
| KSFS 6 Weeks | 62.2 ± 18.1 | 41.2 ± 27.2 | <0.001 (-25.1, -11.4)* |
| KSS 6 Weeks | 87.3 ± 10.9 | 89.4 ± 7.9 | 0.252 (-1.5, 5.8) |
| KSFS 3 Months | 74.8 ± 14.7 | 63.4 ± 21.6 | 0.006 (-19.3, -3.4)* |
| KSS 3 Months | 91.0 ± 9.6 | 91.7 ± 6.4 | 0.697 (-2.9, 4.4) |
| KSFS 6 Months | 78.1 ± 14.6 | 77.9 ± 15.9 | 0.946 (-6.8, 6.3) |
| KSS 6 Months | 93.1 ± 8.8 | 94.7 ± 6.6 | 0.356 (-1.9, 5.2) |
| KSFS 12 Months | 82.2 ± 14.8 | 77.4 ± 20.8 | 0.160 (-11.5, 1.9) |
| KSS 12 Months | 94.7 ± 5.7 | 96.8 ± 4.2 | 0.037 (0.1, 4.1)* |
| Difference | -0.91 ± 2.12 | -0.40 ± 3.09 | 0.272 (-0.4, 1.4) |
| Absolute Difference | 1.85 ± 1.36 | 2.11 ± 2.28 | 0.488 (-0.5, 1.0) |

Supplementary Table 5. Subgroup Analysis Sex – Student’s T-Test

|  | Robotic-Assisted | ABN | P-value (95% CI) |
| --- | --- | --- | --- |
| Female - Mean ± SD | (n=84) | (n=39) |  |
| KSFS 6 Weeks | 56.8 ± 18.0 | 40.2 ± 14.2 | 0.014 (-23.9, -2.8)* |
| KSS 6 Weeks | 88.5 ± 7.2 | 56.8 ± 18.0 | 0.908 (-3.4, 3.8) |
| KSFS 3 Months | 72.9 ± 14.2 | 65.3 ± 15.3 | 0.029 (-14.3, -0.8)* |
| KSS 3 Months | 91.9 ± 8.1 | 91.6 ± 6.9 | 0.851 (-3.8, 3.1) |
| KSFS 6 Months | 76.6 ± 14.3 | 79.4 ± 12.0 | 0.350 (-3.1, 8.6) |
| KSS 6 Months | 94.7 ± 4.7 | 94.7 ± 6.5 | 0.974 (-2.3, 2.3) |
| KSFS 12 Months | 82.1 ± 14.2 | 79.6 ± 20.2 | 0.459 (-9.2, 4.2) |
| KSS 12 Months | 95.6 ± 4.3 | 95.8 ± 4.9 | 0.760 (-1.5, 2.1) |
| Difference | -0.96 ± 1.88 | 0.18 ± 3.32 | 0.050 (0.0, 2.3) |
| Absolute Difference | 1.68 ± 1.28 | 2.08 ± 2.57 | 0.364 (-0.5, 1.3) |
| Male - Mean ± SD | (n=28) | (n=27) |  |
| KSFS 6 Weeks | 69.6 ± 17.3 | 52.6 ± 24.2 | 0.011 (-29.8, -4.2)* |
| KSS 6 Weeks | 88.4 ± 10.8 | 91.0 ± 7.3 | 0.338 (-2.9, 8.3) |
| KSFS 3 Months | 83.3 ± 12.6 | 65.3 ± 29.1 | 0.018 (-32.6, -3.4)* |
| KSS 3 Months | 89.8 ± 14.2 | 92.9 ± 4.6 | 0.432 (-5.0, 11.1) |
| KSFS 6 Months | 83.3 ± 20.8 | 77.1 ± 24.3 | 0.388 (-20.7, 8.2) |
| KSS 6 Months | 90.9 ± 14.1 | 94.7 ± 5.4 | 0.275 (-3.2, 10.8) |
| KSFS 12 Months | 83.8 ± 16.7 | 73.8 ± 26.4 | 0.113 (-22.7, 2.5) |
| KSS 12 Months | 95.1 ± 3.9 | 83.8 ± 16.7 | 0.196 (-1.3, 6.1) |
| Difference | -0.07 ± 2.28 | -1.22 ± 3.18 | 0.128 (-2.6, 0.3) |
| Absolute Difference | 1.71 ± 1.46 | 2.63 ± 2.11 | 0.067 (-0.1, 1.9) |
